# 

**Published:** 1891-10

**Authors:** 


### CONTENTS:

Article I.—Is Dentistry a Dangerous Profession. By Dudley W. Buxton, M. D. - - - - - - - 241

" II.—Twenty-third Annual Session of the Georgia State Dental Society. - - - - - - - 251

" III.—Bacteria of the Human Mouth. By Dr. E P. Beadles. 261

" IV.—Caries. By A. J. Holmes, D. D. S., L. D. S. - - 271

" V.—The Care of the Deciduous Teeth. By J. E. Hinkins. 277

" VI.—Third Dentition. - - - - - - 283

### COMMUNICATIONS.

A Correction. - - - - - - - - 284

Maryland State Dental Association. - - - - - 285

### MONTHLY SUMMARY.

Insanity following Extraction of Nineteen Teeth. - - - 285

No More Artificial Teeth. - - - - - - - - 286

To Sterilize Instruments without Dulling Them. - - - 287

Dermatol. - - - - - - - - - - 287

Aseptinic Acid. - - - - - - - - 288

When the Brain is Idle. - - - - - - - 288

